# Laminin α4 overexpression in the anterior lens capsule may contribute to the senescence of human lens epithelial cells in age-related cataract

**DOI:** 10.18632/aging.101943

**Published:** 2019-05-10

**Authors:** Yu Yan, Haiyang Yu, Liyao Sun, Hanruo Liu, Chao Wang, Xi Wei, Fanqian Song, Hulun Li, Hongyan Ge, Hua Qian, Xiaoguang Li, Xianling Tang, Ping Liu

**Affiliations:** 1 Eye Hospital, First Affiliated Hospital, Harbin Medical University, Harbin 150001, China; 2 Department of Pharmacology, College of Pharmacy, Harbin Medical University, and Heilongjiang Academy of Medical Sciences, Harbin 150081, China; 3 Beijing Institute of Ophthalmology, Beijing Tongren Hospital, Capital Medical University, Beijing Ophthalmology & Visual Science Key Lab, Beijing 100000, China; 4 Department of Neurobiology, Neurobiology Key Laboratory, Harbin Medical University, Harbin 150081, China

**Keywords:** age-related cataract, anterior lens capsule, laminin α4, human lens epithelial cell, senescence, basement membrane

## Abstract

Senescence is a leading cause of age-related cataract (ARC). The current study indicated that the senescence-associated protein, p53, total laminin (LM), LMα4, and transforming growth factor-beta1 (TGF-β1) in the cataractous anterior lens capsules (ALCs) increase with the grades of ARC. In cataractous ALCs, patient age, total LM, LMα4, TGF-β1, were all positively correlated with p53. In lens epithelial cell (HLE B-3) senescence models, matrix metalloproteinase-9 (MMP-9) alleviated senescence by decreasing the expression of total LM and LMα4; TGF-β1 induced senescence by increasing the expression of total LM and LMα4. Furthermore, MMP-9 silencing increased p-p38 and LMα4 expression; anti-LMα4 globular domain antibody alleviated senescence by decreasing the expression of p-p38 and LMα4; pharmacological inhibition of p38 MAPK signaling alleviated senescence by decreasing the expression of LMα4. Finally, in cataractous ALCs, positive correlations were found between LMα4 and total LM, as well as between LMα4 and TGF-β1. Taken together, our results implied that the elevated LMα4, which was possibly caused by the decreased MMP-9, increased TGF-β1 and activated p38 MAPK signaling during senescence, leading to the development of ARC. LMα4 and its regulatory factors show potential as targets for drug development for prevention and treatment of ARC.

## Introduction

Age-related cataract (ARC), characterized by lens opacity and visual impairment in the middle-aged and elderly, is responsible for nearly half of all blindness worldwide [1]. Previous studies have suggested that various risk factors, including age, sex, social status, ultraviolet radiation, smoking, and diabetes mellitus, may contribute to the development of ARC [2,3]. However, at present, mechanisms underlying the pathology of ARC remain unclear. Aside from surgery, there is a lack of effective treatments for curing ARC [3,4].

Oxidative stress caused by reactive oxygen species (ROS) has long been recognized as a major mechanism by which cells are damaged and cataracts are formed [5–7]. Hydrogen peroxide (H_2_O_2_) is the main intracellular ROS in the aqueous humor that can cause protein oxidation and aggregation, lipid peroxidation, and DNA damage, and can decrease antioxidant levels in the lens, eventually accelerating the damage to the lens epithelial cells (LECs), resulting in subsequent cataract development [8–10]. With age, the lens undergoes several morphological, biochemical and physical changes [11], wherein thickness of the lens capsule as well as the accumulation of advanced glycation end products in the lens capsule increased [12–14], all of which may cause for the formation of ARC. Recent studies have reported that more senescent LECs were observed in the elderly ARC patients, thus oxidative stress induced cellular senescence may contribute to the development of ARC [15,16].

The lens capsule is a modified basement membrane (BM) that completely surrounds the ocular lens. The normal lens capsule is mainly composed of laminin (LM) and type IV collagen [17–19]. Type I collagen is not detected in the capsule of normal lenses [20]. However, Type I collagen is expressed in cataractous lenses [21,22], and increases with age in human lens capsules [23]. LMs are heterotrimers of α, β, and γ subunits drawn from a total of 5α, 3β, and 3γ isoforms [24,25]. LM, which is the first BM component to appear during the early stages of embryonic development, promotes cell proliferation, migration, and differentiation [26,27]. In aged tissues, LM expression was decreased [28–30]; however, other studies have indicated that it was increased [31]. LM was highly expressed in the capsules of cataractous lenses [32,33]. Previous studies have demonstrated that the human adult lens capsule is composed of LMα5-α1, LMβ2-β1, and LMγ1 subunits [34]. The relationship between LM subunits and senescence was well studied in cells aside from LECs. One of our recent studies revealed that LMα2, LMα1, and LMγ1 were increased in senescent corneal endothelial cells [35]. LMβ1 was upregulated in senescent cardiac endothelial cells, while LMβ2 was downregulated [36]. LMγ1 and LMβ2 were found to be increased in the senescent cerebral vessels [37]. LMα4 knockout mice displayed a senescent phenotype in skeletal neuromuscular junctions [38]. LMα4 localization pattern was changed in senescent skeletal neuromuscular junctions, but its expression level was not reduced [39]. However, what LM subunits and how they contribute to the formation of cataract remains unclear.

Matrix metalloproteinase-9 (MMP-9), a proteolytic enzyme, has been implicated in the progression of various kinds of cataracts, including anterior subcapsular cataract [40], posterior capsular opacification [41] and UVB-induced cataract [42]. MMP-9 could process LM and latent transforming growth factor-beta (TGF-β). It promotes cell survival by degrading LMs in neuronal cells [43]. Furthermore, it increases the activation and transcription of TGF-β1 during cardiac aging [44]. In addition, numerous LM peptides were able to induce MMP-9 expression [45,46]. LECs cultured on type I collagen-coated dishes exhibited high expression levels of the pro-form of MMP-9 [20]. However, there is currently no evidence that shows a potential role of MMP-9 in senescent LECs or in senescent lens capsules of ARC.

TGF-β1 is involved in cell proliferation, migration, differentiation, and apoptosis as well as extracellular matrix accumulation in various cells [47,48]. It is present in the aqueous humor and vitreous, and is responsible for the induction of cataract and cell senescence at a relatively high concentration [49–53]. TGF-β1 was highly expressed in LECs with kinds of cataracts, such anterior subcapsular cataract [54], posterior capsular opacification [55], and diabetic cataract [56,57], and was found to increase with age [58,59]. TGF-β1 downregulated the mRNA level of total LM in apoptotic HLE B-3 cells [60], and induced aberrant deposition of LMα2-α1 and LMβ1 in glomerular BMs [61]. It also may stimulate the expression of type I collagen in bovine lens epithelial explants and intact rabbit lens [62]. However, there is currently no evidence showing a potential role of TGF-β1 in senescent LECs or senescent lens capsules of ARC.

Na+/K+-ATPase is an important membrane ion transporter existing in all mammalian cells. Aside from the ion transporting function, Na+/K+-ATPase related signaling has been demonstrated to play a role in the regulation of cell growth, survival and differentiation [63]. Reduced activity of Na+/K+-ATPase in cataractous lens cells, led to overhydration, protein loss, and ionic imbalance [64]. Decreased Na+/K+-ATPase expression was observed in senescent corneal endothelial cells [65]. Na+/K+-ATPase α1 (ATP1A1), which is the α1 subunit of Na+/K+-ATPase, was expressed more in mature (differentiated) lens fiber cells and equatorial LECs, but hardly at all in differentiating zone lens fibers [66,67]. ATP1A1 expression was increased in senescent opossum kidney cells [68].

As a major intracellular ROS of the ocular lens, H_2_O_2_ has been used as the most common inducer of cataract as well as LEC senescence [69–71]. Senescent cells were defined as irreversibly growth arrested cells displaying distinctive morphological changes, including a flattened appearance, higher granularity, size enlargement, and altered nucleus to cytoplasm ratios [72–74]. Moreover, higher senescence-associated-galactosidase (SA-β-gal) activities as well as higher expression levels of senescence molecular markers, such as p21, p53, and lysosomal β-galactosidase (GLB1), were observed in senescent cells [75,76]. In our previous studies, we demonstrated that a human LEC line (HLE B-3) could produce an anterior-capsule-like BM with rich LMs. This anterior-capsule-like material may be used to study the contribution of BM proteins, such as LMs, to cataract pathogenesis [77]. Thus, the current study explored the role of LMs in the formation of ARC using H_2_O_2_-induced HLE B-3 senescent cells and senescent cell BMs.

In this study, we explored the pathogenesis of ARC. We focused on total LM, particularly LMα4 expression, and their potential mediators MMP-9 and TGF-β1, in ALCs of ARC. Additionally, in H_2_O_2_-induced HLE B-3 senescent cell models, we studied the effect of MMP-9, TGF-β1, and LMα4 on cell senescence, and elucidated oxidative stress-mediated signaling pathways underlying LMα4 deposition in ALCs of ARC. Our data provided new targets for drug development for prevention and treatment of ARC.

## RESULTS

### Senescence in ALCs increased with the grades of ARC

**Figure 1:**
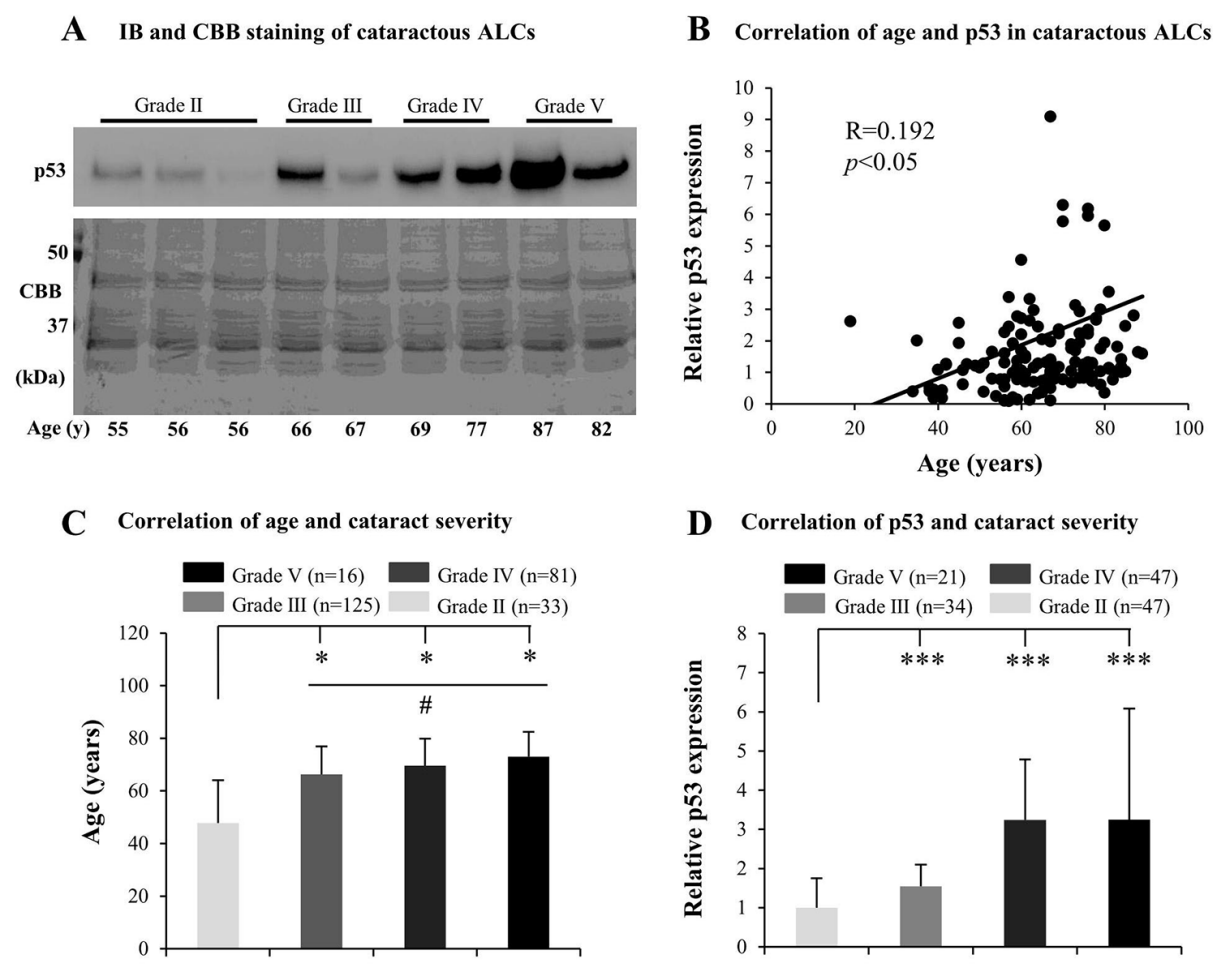
**Senescence associated markers increase with the grades of age-related cataract (ARC) in human anterior lens capsules (ALCs).** Human ALCs graded before surgery for cataract severity using the Emery-Little Classification System of nuclear opacity grade were obtained from the anterior surface of cataractous lenses during surgery. **(A)** Photographs for representing immunoblot analysis of p53 and corresponding Coomassie brilliant blue (CBB) staining in cataractous ALCs of different grades and ages. **(B)** Relative expression levels of p53 immunoblots in cataractous ALCs. Quantification of immunoblots was processed using Image J. The figure depicts Pearson correlation between age and p53 protein expression (n = 144). **(C)** The correlation between patient age and ARC grades (Data were analyzed via One-way ANOVA). **(D)** The correlation between relative p53 expression and ARC grades (Data are analyzed via Wilcoxon Rank Sum Test). Data were shown as mean ± SD. *, *p*<0.05; **, *p*<0.01; ***, *p*<0.001 versus grade II group. #, *p*<0.05 versus grade III group.

Many mechanisms underlying ARC, including the effect of aging, have been investigated, but the association between senescence and the grades of cataract remains unclear. We inferred that ALC senescence may contribute to the development of ARC. Therefore, we initially tested senescence-related markers, including p53, p21, and GLB1, in cataractous ALCs. We found that the protein levels of p53 in elderly cataractous ALCs were elevated (Fig. 1A), in an age-dependent manner (R=0.192) (Fig. 1B), as evidenced via immunoblot analysis and Coomassie brilliant blue (CBB) staining. However, expression of GLB1 and p21 in the elderly cataractous ALCs were not elevated (data not shown). We further found that the age of ARC patients and protein levels of p53 increased with increasing ARC grades. The ages of patients in grades V, IV and III were all significantly higher than that in grade II (*p*<0.05) (Fig. 1C). The age of patients in grade V were also higher than that in grade III (*p*<0.05) (Fig. 1C). The protein levels of p53 in cataractous ALCs of grades V, IV and III were all much higher than that in grade II (*p*<0.001) (Fig. 1D). These results suggested that increased senescence in cataractous ALCs was associated with the grades of ARC.

### Elevated LMs in cataractous ALCs with senescence

**Figure 2:**
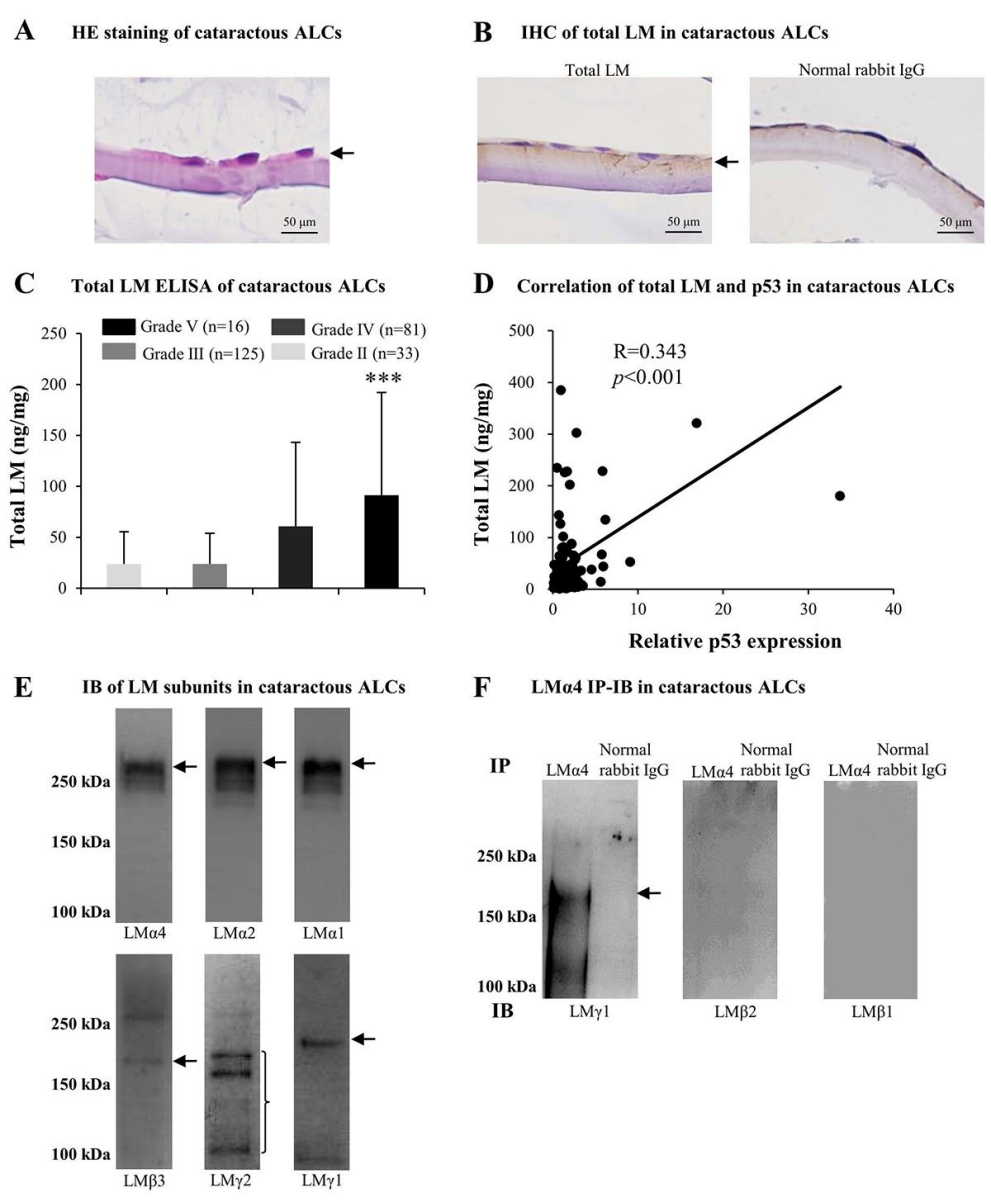
**Excess laminins (LMs) in the senescent anterior lens capsules (ALCs) of age-related cataract (ARC).** Representative photographs of hematoxylin and eosin (HE) staining of cataractous ALCs **(A)** and immunohistochemistry (IHC) of LMs in cataractous ALCs **(B)** (Scale bars: 50 μm). **(C)** Total LM in cataractous ALCs with different grades as detected by ELISA (Data were shown as mean ± SD and were analyzed by one-way ANOVA). ***, *p*<0.001 versus grade II group. The figure depicts Pearson correlation between total LM expression and relative p53 expression (n = 128) **(D)**. **(E)** Immunoblot analysis of LM subunits in mixed cataractous ALCs (n = 10). **(F)** LM trimers in mixed cataractous ALCs as detected by LMα4. Immunoprecipitation-immunoblotting (IP-IB) using antibodies against LMα4 to precipitate LM trimers and antibodies against LMγ1, LMβ2 and LMβ1 for IB (n = 10).

Based on the above findings regarding cataractous ALCs, we studied factors contributing to senescence. Much evidence indicated that LMs were involved in cataract formation as well as in cellular senescence. Therefore, we analyzed LM expression in ALCs of ARC. The distribution of LMs in cataractous ALCs was analyzed using HE staining and immunohistochemistry (IHC). LMs were primarily distributed in the ALC layer closest to the LECs (Figs. 2A and 2B). ELISA indicated that the expression of total LM in cataractous ALCs increased with increasing ARC grades, and that total LM in ALCs of grade V was significantly higher than that of grade II (*p*<0.001); (Fig. 2C). Furthermore, the total LM protein level via ELISA was increased in a senescence-dependent manner (R=0.343); (Fig. 2D). To further investigate which LM subunit(s) or LM trimer(s) in ALCs were involved in ARC development, extracts of mixed cataractous ALCs were examined via immunoblotting (IB) and immunoprecipitation-immunoblotting (IP-IB). Cataractous ALCs were positive for 6 of 11 LM subunits, including LMα4, α2, α1, β3, γ2, and γ1 (Fig. 2E). Immunoprecipitates of cataractous ALCs obtained using antibodies against LMα4 were positive for LMγ1, but negative for LMβ1 and LMβ2 (Fig. 2F), indicating the presence of LM411 or LM421 trimers in cataractous ALCs. However, immunoprecipitates of cataractous ALCs obtained via antibodies against LMα2 or LMα1 were negative for all β and γ subunits of LMs (data not shown).

Combined with literature reviews, these results suggested that the increase of total LM in cataractous ALCs, which displaying ARC severity, may be associated with increased senescence. In ALCs, LMs, particularly LM411 or LM421 trimer, or both, may participate in the pathogenesis of ARC during senescence.

### Premature senescence model of LECs induced by H_2_O_2_

**Figure 3:**
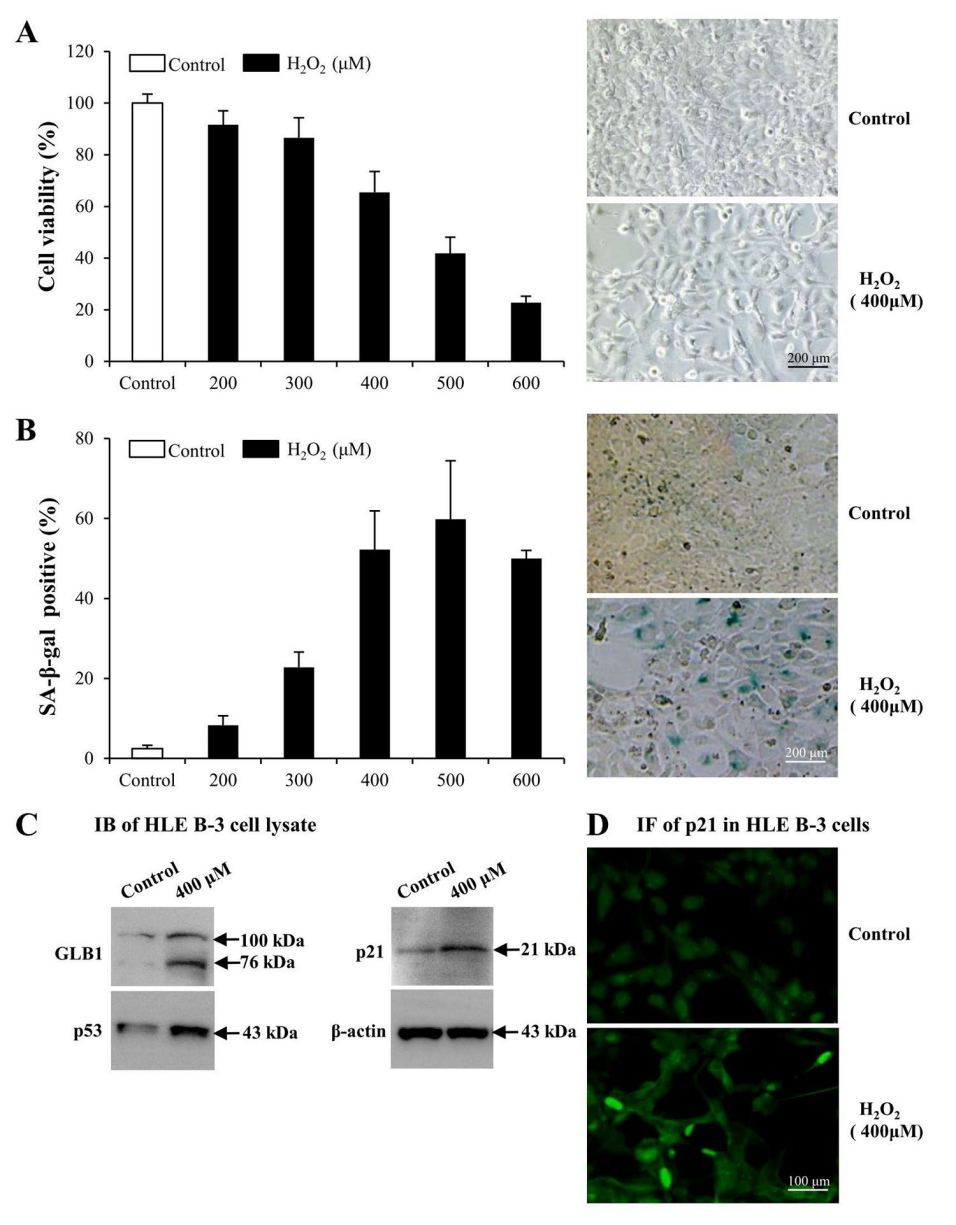
**Premature senescence model of human lens epithelial cells (HLE B-3) induced by H_2_O_2_.** Cells of the control group were cultured in medium only, whereas cells of senescent group were cultured in medium with H__2__O__2__ for 96 h. **(A)** Viabilities (left) of HLE B-3 cells treated with different concentrations of H__2__O__2__ (0–600 μM) for 96 h, as measured via an MTT assay. Morphologic changes (right) of HLE B-3 cells following a 96 h exposure to 400 μM H__2__O__2__. **(B)** Percentage of SA-β-gal-positive cells in HLE B-3 cells treated with different concentrations of H__2__O__2__ (0–600 μM) (left). SA-β-gal activity as measured by cell staining (right). **(C)** Immunoblot analysis of GLB1, p21 and P53 in HLE B-3 cells. **(D)** Immunofluorescence analysis of p21 (green) in HLE B-3 cell nuclei. (Scale bars: 100 μm). Data were shown as mean ± SD and are representative of 3 independent experiments.

To determine whether LMs, particularly LM411 and LM421 trimers in ALCs, are involved in the pathogenesis of ARC in cellular senescence, we used senescent human LEC (HLE B-3) as models. We exposed cultured HLE B-3 cells to different H_2_O_2_ concentrations to stimulate oxidative stress associated cell senescence. H_2_O_2_ impaired cell viability in a dose dependent manner (Fig. 3A, left). Exposure to 400 μM H_2_O_2_, cell viability was decreased to 65% of that of the control. Furthermore, the morphology of H_2_O_2_ treated cells showed features of senescent cells, such as being large and flattened and containing accumulations of granular cytoplasmic inclusions (Fig. 3A, right). Increased senescence-associated β-galactosidase (SA-β-gal) activity has been widely used as an in vitro biomarker for cellular senescence [73,74]. Treatment with various H_2_O_2_ concentrations caused a significant increase in the percentage of SA-β-gal-positive cells in HLE B-3 cells, and the SA-β-gal positive cell percentage in HLE B-3 cells treated with 400 μM of H_2_O_2_ was approximately 52% (Fig. 3B).

As reported, increased expression of GLB1, p53 and p21 are potential biomarkers of cell senescence [75,76]. As indicated via IB, expression levels of GLB1, p53 and p21 in HLE B-3 cells treated with 400 μM of H_2_O_2_ were significantly higher than those in untreated HLE B-3 cells (Fig. 3C). Immunofluorescence (IF) demonstrated that p21 expression in nuclei of HLE B-3 cells treated with 400 μM H_2_O_2_ was much stronger than that in nuclei of untreated HLE B-3 cells (Fig. 3D).

The abovementioned observations suggested that many changes took place in HLE B-3 cells following prolonged exposure to 400 μM H_2_O_2_. In addition to senescent-like morphological changes, other senescent cells characteristics, such as the enhancement of SA-β-gal activity and the overexpression of 3 senescence biomarkers, GLB1, p53 and p21 in HLE B-3 cells, were observed. Therefore, as these cells may mimic senescent LECs *in vivo*, HLE B-3 cells treated with 400 μM H_2_O_2_ for 96 h were used as senescent cells for the studies which followed.

### Elevated LMs in senescent BMs induced by H_2_O_2_

**Figure 4:**
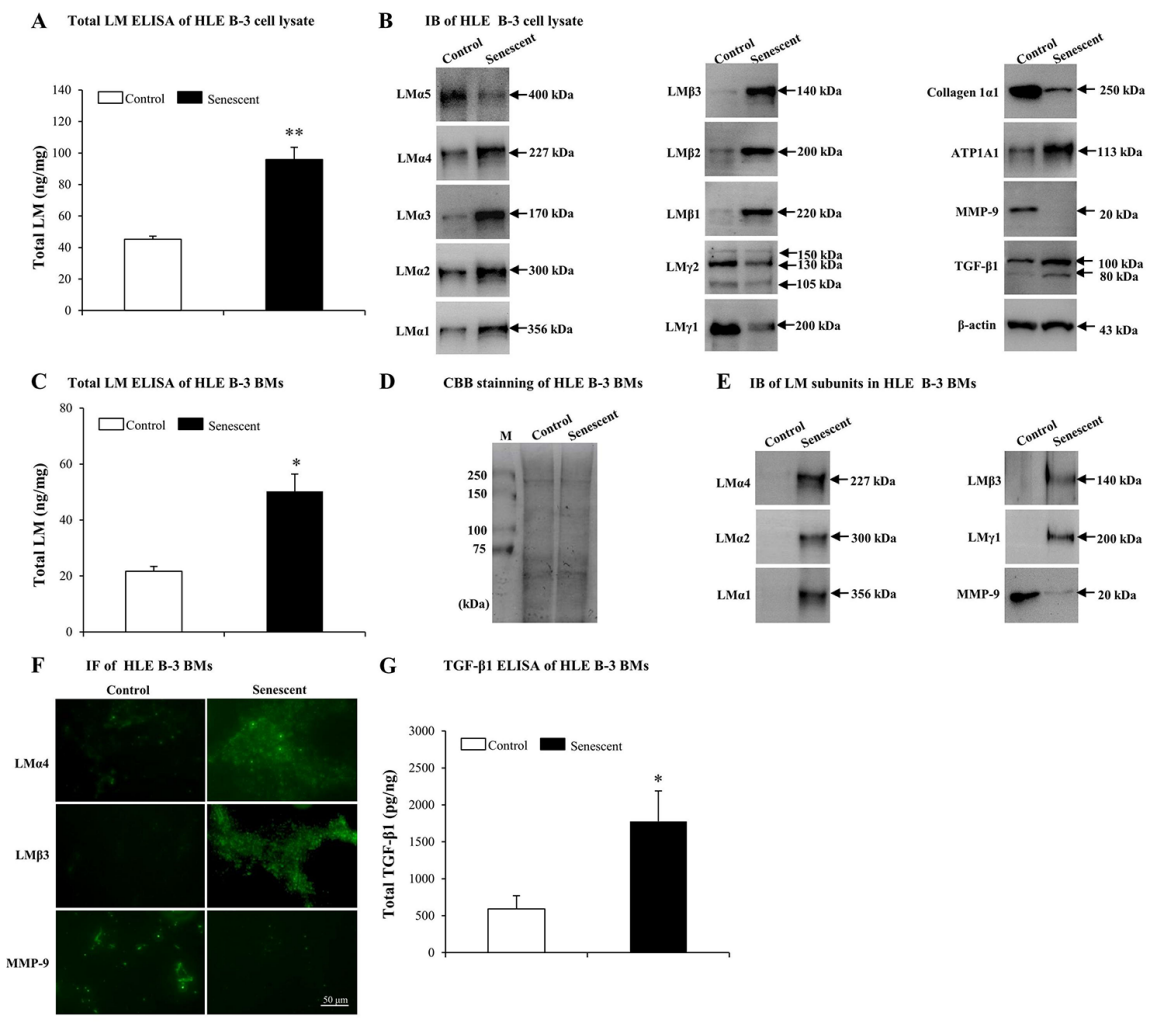
**Elevated laminins (LMs) in senescent HLE B-3 cells and cell basement membranes (BMs) induced by H_2_O_2_.** Cells (or cell BMs) of the control group were cultured in medium only, while cells (or cell BMs) of senescent group were cultured in medium with H__2__O__2__ (400 μM) for 96 h. **(A)** Total LM in HLE B-3 cells as detected by ELISA (data were analyzed by paired t-test). **(B)** Immunoblot analysis of 11 LM subunits, collagen 1α1, ATP1A1, MMP-9, and TGF-β1 in HLE B-3 cells. **(C)** Total LM in HLE B-3 cell BMs as detected by ELISA (data were analyzed by paired t-test). **(D)** SDS-PAGE analysis followed by CBB staining of HLE B-3 cell BMs. **(E)** Immunoblot analysis of LM subunits and MMP-9 in HLE B-3 cell BMs. **(F)** Immunofluorescence analysis of LMα4, LMβ3, and MMP-9 in HLE B-3 cell BMs (Scale bars: 50 μm). **(G)** TGF-β1 in HLE B-3 cell BMs, as detected by ELISA (data were analyzed using One-way ANOVA). Data are shown as mean ± SD. *, *p*<0.05; **, *p*<0.01 versus control group.

In order to determine whether LM expression was different between senescent and normal HLE B-3 cells, we tested LMs in HLE B-3 cell lysate and cell BMs via ELISA and IB. In senescent HLE B-3 cell lysate, compared to the control, ELISA results indicated that total LM expression showed an increase (*p*<0.01); (Fig. 4A). IB indicated that, LMα4, α3, α2, α1, β3, β2, β1, and ATP1A1 increased, while expression levels of LMα5, γ2 and γ1 subunits and collagen 1α1 decreased (Fig. 4B). To elucidate potential molecular mechanisms involved in inducing changes of LMs, ATP1A1 and collagen 1α1 in senescent HLE B-3 cells, we analyzed MMP-9 and TGF-β1 using IB. The results showed that senescent HLE B-3 cells displayed a lower MMP-9 expression level and a higher TGF-β1 expression level, compared to those of the control (Fig. 4B).

ELISA indicated that senescent cell BMs showed higher total LM expression levels, compared to those of the control (*p*<0.05); (Fig. 4C). HLE B-3 cell BMs of control and senescent groups showed the same polypeptide bands intensities via CBB staining (Fig. 4D). IB indicated that, compared to those of the control, senescent cell BMs showed higher expression levels of LMα4, LMα2, LMα1, LMβ3, LMγ1 and collagen 1α1, as well as decreased expression levels of MMP-9 ((Fig. 4E). IF indicated stronger LMα4, LMβ3 expression levels and weaker of MMP-9 expression levels (Fig. 4F). Both IB and IF did not detect any TGF-β1 in cell BMs of both senescent and control HLE B-3 cells (data not shown), whereas, ELISA showed increased total TGF-β1 expression levels in senescent cell BMs compared those in the control (*p*<0.05); (Fig. 4G).

Elevated expression of total LM in senescent BMs of HLE B-3 cells, confirmed the involvement of LMs in senescence. Increasingly, evidence indicates that MMP-9 silencing and TGF-β1 may accelerate senescence. In our above results, senescent HLE B-3 cells and cell BMs were shown decreased MMP-9 and increased TGF-β1 expressions. Considering these changes, we hypothesized that senescent-associated accumulation of LMs in cataractous ALCs may be medicated by MMP-9 and TGF-β1.

### MMP-9 reduces the cell senescence and LM deposition induced by H_2_O_2_

**Figure 5:**
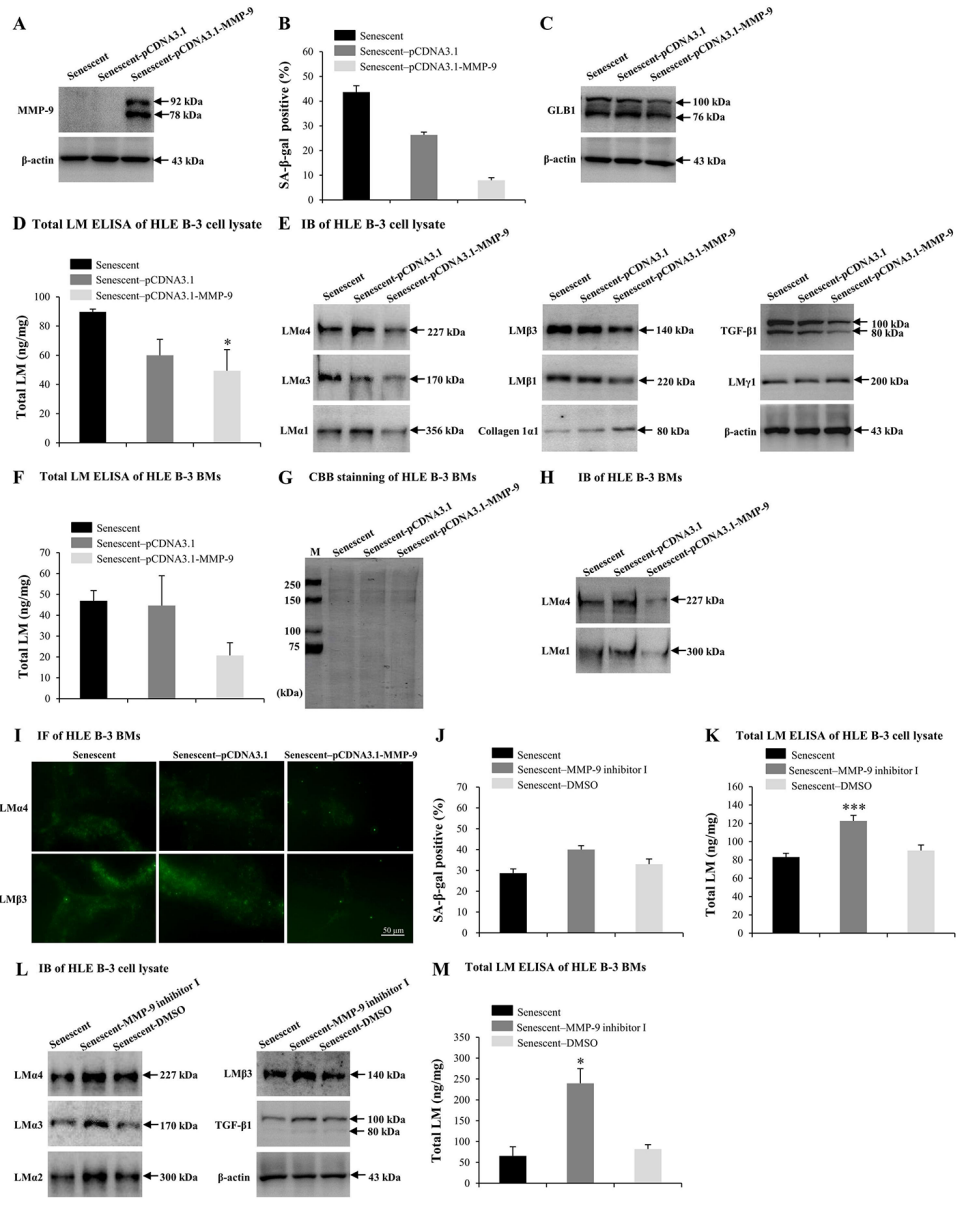
**MMP-9 reduces cell senescence and laminin (LM) deposition induced by H_2_O_2_.** Senescent HLE B-3 cells [or cell basement membranes (BMs)] were cultured in medium with H__2__O__2__ (400 μM) for 96 h. **(A-I)** HLE B-3 cells were treated with H__2__O__2__ only, or in combination with indicated plasmid. **(A)** Immunoblot analysis of MMP-9 in HLE B-3 cells. **(B-C)** Percentage of SA-β-gal-positive cells **(B)** and protein expression of GLB1 **(C)** in HLE B-3 cells. **(D)** Total LM in HLE B-3 cells, detected by ELISA. **(E)** Protein expressions of TGF-β1 and LM subunits in HLE B-3 cells, detected by IB. **(F)** Total LM in HLE B-3 cell BMs, detected by ELISA. **(G)** SDS-PAGE analysis followed by CBB staining of HLE B-3 cell BMs. **(H)** Immunoblot analysis of LMα4 and LMα1 in HLE B-3 cell BMs. **(I)** Immunofluorescence analysis of LMα4 (green) and LMβ3 (green) in HLE B-3 cell BMs (Scale bars: 50 μm). **(J-M)** HLE B-3 cells were treated with H__2__O__2__ only, or in combination with indicated siRNA. **(J)** The percentage of SA-β-gal-positive cells in HLE B-3 cells. **(K)** Total LM in HLE B-3 cells, as detected by ELISA. **(L)** Immunoblot analysis of LM subunits and TGF-β1 in HLE B-3 cells. **(M)** Total LM in HLE B-3 cell BMs, as detected by ELISA. Data are shown as mean ± SD and were analyzed using paired t-test. *, *p*<0.05; ***, *p*<0.001.

Numerous studies have reported that MMP-9 may participate in LM degradation [78,79]. MMP-9-deficient mice displayed reduced retinal ganglion cells loss as a result of limited LM degradation [79]. Therefore, we attempted to determine the role of excess MMP-9 in LM response to senescence. Senescent HLE B-3 cells were transfected with the MMP-9 plasmid. IB demonstrated that senescent HLE B-3 cells transfected with pCDNA3.1-MMP-9 showed increased MMP-9 expression levels (Fig. 5A), decreased percentage of senescent cells (Fig. 5B) and decreased GLB1 expression levels (Fig. 5C), compared with those of the blank vector (control), additionally, ELISA results suggested a decrease in total LM expression levels (*p*<0.05) (Fig. 5D), IB results showed decreased LMα4, LMα3, LMα1, LMβ3, LMβ1, and TGF-β1 levels, increased collagen 1α1 levels, and similar LMγ1 expression levels (Fig. 5E). Compared to the blank vector control, BMs of senescent HLE B-3 cells transfected with pCDNA3.1-MMP-9 showed decreased levels of total LM as indicated by ELISA, although these results were not statistically significant (*p*=0.102); (Fig. 5F). CBB staining showed that HLE B-3 senescent cell BMs of the control, blank vector control, and the pCDNA3.1-MMP-9 group displayed similar polypeptide band intensities (Fig. 5G). Compared to blank vector control, BMs of senescent HLE B-3 cells transfected with pCDNA3.1-MMP-9 showed decreased LMα4 and LMα1 (Fig. 5H) and LMα4 and LMβ3 (Fig. 5I) expression levels, as indicated by IB and IF, respectively.

MMP-9 inhibitor was employed to further confirm the effects of MMP-9 on cell senescence and LM deposition in the BMs of HLE-B-3 cells. Compared to solution control, senescent HLE B-3 cells treated with MMP-9 inhibitor I showed increased senescent cell percentages (Fig. 5J), increased total LM expression levels by ELISA (*p*<0.001); (Fig. 5K), as well as increased LMα4, LMα3, LMα2, LMβ3 and TGF-β1 by IB (Fig. 5L). Compared to solution control, BMs of senescent HLE B-3 cells treated with MMP-9 inhibitor I showed increased total LM expression levels by ELISA (*p*<0.05); (Fig. 5M). These results confirmed that MMP-9 may reduce senescence of HLE B-3 cells by degrading the abnormal deposition of total LM and LMα4 in senescent BMs.

### TGF-β1 enhances the cell senescence and LM deposition induced by H_2_O_2_

**Figure 6:**
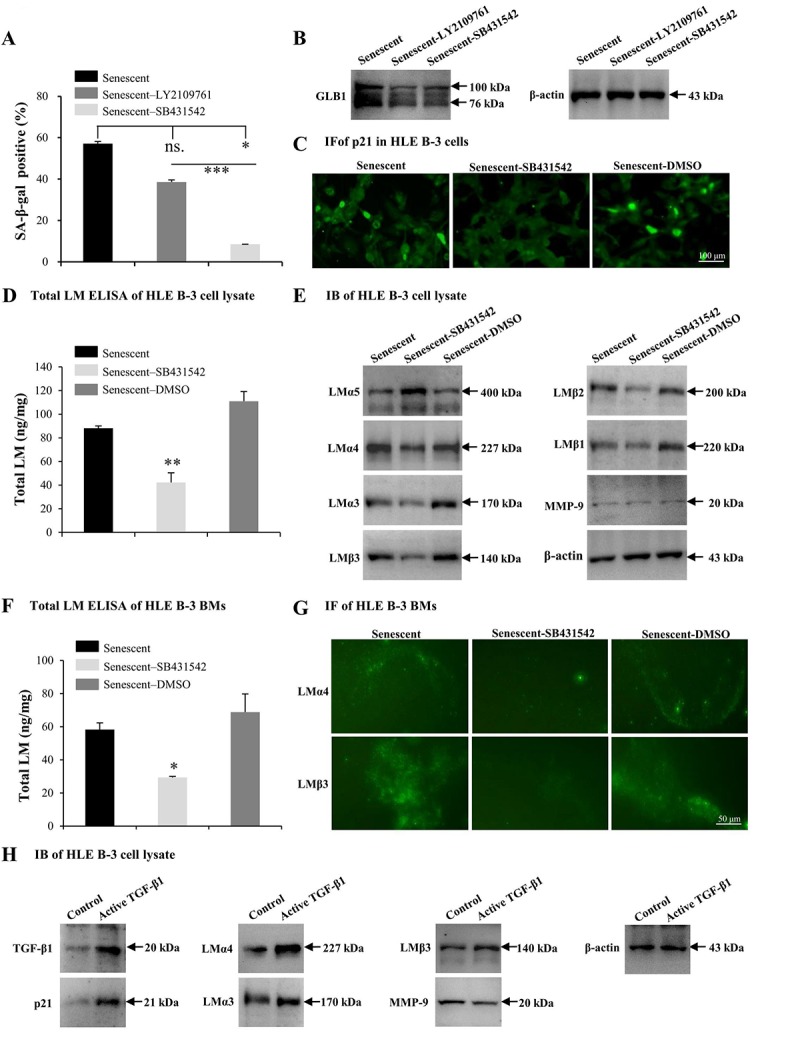
**TGF-β1 enhances cell senescence and laminin (LM) deposition induced by H_2_O_2_.** Senescent HLE B-3 cells [or cell basement membranes (BMs)] were cultured in medium with H__2__O__2__ (400 μM) for 96 h. **(A-G)** HLE B-3 cells were treated with H__2__O__2__ only, or in combination with LY2109761 (5 µM) or SB431542 (10 µM) for 72 h. **(A-C)** Percentage of SA-β-gal-positive cells **(A)**, protein expression of GLB1 **(B)** and immunofluorescence analysis of p21 **(C)** in HLE B-3 cells. **(D)** Total LM in HLE B-3 cells, as detected by ELISA. **(E)** Immunoblot analysis of LM subunits and MMP-9 in HLE B-3 cells. **(F)** Total LM in HLE B-3 cell BM, as detected by ELISA. **(G)** Immunofluorescence analysis of LMα4 (green) and LMβ3 (green) in HLE B-3 cell BMs (Scale bars: 100 μm). **(H)** HLE B-3 cells treated with TGF-β1 (15 ng/ml) for 96 h. Protein expression levels of TGF-β1, p21, MMP-9 and LM subunits in HLE B-3 cells analyzed via IB. Data were shown as mean ± SD and were analyzed using paired t-test. *, *p*<0.05; **, *p*<0.01.

TGF-β1 has been found to induce abnormal deposition of LMs in BMs [61]. In this study, TGF-β1 was increased in HLE B-3 senescent cells and cell BMs. Therefore, the effect of TGF-β1 on senescence and deposition of LMs in HLE B-3 cells and cell BMs was assessed. In the following experiments, SB431542 (TGF-β RI inhibitor), LY2109761 (TGF-β RI/II inhibitor) and human active TGF-β1 recombinant proteins (TGF-β1 RP) were employed. Compared to control senescent cells, senescent HLE B-3 cells treated with SB431542 (*p*<0.05) or LY2109761 (*p*=0.085) displayed a decreased senescent cell percentage (Fig. 6A), and decreased GLB1 expression levels as indicated by IB (Fig. 6B). The anti-senescent effect of SB431542 was stronger than that of LY2109761 (*p*<0.001); (Fig. 6A), thus SB431542 was used in the experiments that followed. Compared to the control solution, senescent HLE B-3 cells treated with SB431542 showed a p21 expression level in the nuclei, as shown by IF (Fig. 6C), lower total LM expression levels as indicated by ELISA (*p*<0.01); (Fig. 6D), lower LMα4, LMα3, and LMβ3-β1 expression levels, higher LMα5 levels, and similar MMP-9 expression levels as demonstrated by IB (Fig. 6E). Compared to control solution, BMs of senescent HLE B-3 cells treated with SB431542 showed decreased total LM expression levels by ELISA (*p*<0.05); (Fig. 6F), weaker LMα4 and LMβ3 expression levels as indicated by IF (Fig. 6G).

We also treated HLE B-3 cells with TGF-β1 RP. IB showed that cells treated with TGF-β1 RP showed higher expression levels of TGF-β1 and p21, LMα4, LMα3 and LMβ3 subunits, as well as lower MMP-9 expression levels, compared to untreated cells (Fig. 6H). These results suggested that TGF-β1 associated signaling may participate in the process of cell senescence, which enhancing cell senescence by increasing the deposition of LM and LMα4 in BMs.

### Interactions between LMα4 and p38 mitogen-activated protein kinase (p38 MAPK) signaling pathway in cell senescence

**Figure 7:**
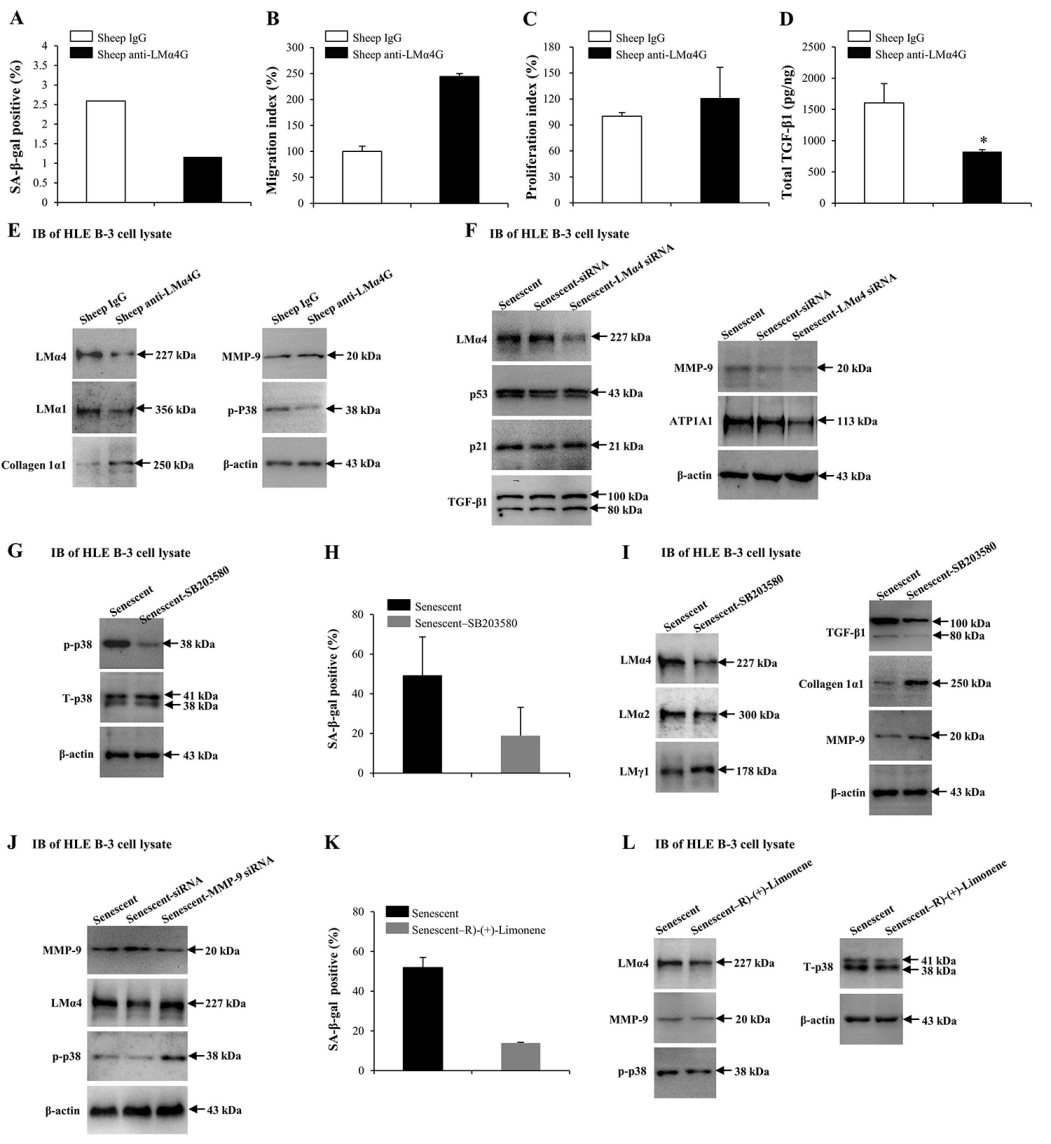
**Interactions between laminin α4 (LMα4) and the activated p38 mitogen-activated protein kinase (p38 MAPK) signaling pathway in cell senescence. (A-E)** Cells were treated with sheep anti-LMα4 globular domain antibodies (2 μg/ml) for 96 h, while cells treated with sheep IgG (2 μg/ml) were selected as the control group. **(A)** Percentage of SA-β-gal-positive cells. **(B)** Migratory abilities of HLE B-3 cells. **(C)** Cell viabilities of HLE B-3 cells measured by CCK-8 assay. **(D)** Total TGF-β1 in HLE B-3 cells detected by ELISA. **(E)** Immunoblot analysis of p-p38, collagen 1α1, MMP-9, and LMs in HLE B-3 cells. **(F)** HLE B-3 cells treated with 400 μM H__2__O__2__ for 96 h only, or in combination with indicated siRNA. Immunoblot analysis of LMα4, p21, p53, TGF-β1, MMP-9 and ATP1A1 in HLE B-3 cells. **(G-I)** HLE B-3 cells treated with H__2__O__2__ (400 μM) only for 96 h, or in combination with SB203580 (30 μM). **(G)** Immunoblot analysis of p-p38 and T-p38 (total p38) in HLE B-3 cells. **(H)** Percentage of SA-β-gal-positive cells. **(I)** Immunoblot analysis of TGF-β1, collagen 1α1, MMP-9 and LMs in HLE B-3 cells. **(J)** HLE B-3 cells were treated with H__2__O__2__ (400 μM) only for 96 h, or in combination with indicated siRNA. Immunoblot analysis of MMP-9, p-p38 and LMα4 in HLE B-3 cells. **(K-L)** HLE B-3 cells were treated with H__2__O__2__ (400 μM) only for 96 h, or in combination with R)-(+)-Limonene (1000 μM). **(K)** Percentage of SA-β-gal-positive cells. **(L)** Immunoblot analysis of p-p38, T-p38, MMP-9 and LMα4 in HLE B-3 cells. Data were shown as mean ± SD and were analyzed using the paired t-test. *, *p*<0.05.

Our results indicated that MMP-9 and TGF-β1 may alter the senescence conditions of HLE B-3 cells, as both regulated the deposition of total LM and LMα4 in senescent BMs. Both sheep anti-LMα4 globular domain antibodies (sheep anti-LMα4G) and small interfering RNA (siRNA) were employed to know the possible role of LMα4 in cell senescence and senescence-associated protein expression changes in HLE B-3 cells. Compared to the control, HLE B-3 cells treated with sheep anti-LMα4G displayed lower senescent cell percentages (Fig. 7A), and higher cell migration abilities (*p*=0.083); (Fig. 7B) and viabilities (*p*=0.170); (Fig. 7C), although these were not statistically significant. Additionally, lower total TGF-β1 expression levels were indicated by ELISA (*p*<0.05); (Fig. 7D), and lower LMα4, LMα1, and p-p38 levels, higher collagen 1α1 expression levels, and similar MMP-9 expression levels, were indicated by IB (Fig. 7E). Compared to siRNA transfection (control), senescent HLE B-3 cells transfected with LMα4 siRNA showed lower LMα4 and ATP1A expression levels, and similar p53, p21, TGF-β1, and MMP-9 expression levels, as demonstrated by IB (Fig. 7F). These results indicated that excess LMα4 contributes to cell senescence.

Cell senescence is typically triggered by the activation of signaling pathways, including the stress-activated p38 MAPK pathway, extracellular signal-regulated kinase (ERK) pathway, and the c-Jun N-terminal kinase (JNK) pathway. Studies showed that p38 MAPK not only played an important role in cellular senescence but was also involved in LM signaling [80–82]. Thus, the p38 MAPK signaling pathway was selected to explore the mechanism underlying the role of LMα4 in senescence. Compared to senescent cells (control), senescent cells treated with SB203580 showed decreased p-p38 and similar total p38 (T-p38) expression levels (Fig. 7G), lowered senescent cell percentages (Fig. 7H), lowered LMα4, LMα2 and TGF-β1 expression levels and higher LMγ1, collagen 1α1 and MMP-9 expression levels, as shown by IB (Fig. 7I). We further investigated whether p38 MAPK proteins participated in MMP-9 related LMα4 up-regulation during senescence. Compared with siRNA (control), senescent HLE B-3 cells transfected with MMP-9 siRNA showed lower MMP-9, and higher LMα4 and p-p38 expression levels, as indicated by IB (Fig. 7J).

In order to further confirm the association between p38 MAPK phosphorylation and LMα4 accumulation in H_2_O_2_-induced cell senescence. HLE B-3 cells were exposed to R)-(+)-Limonene, a potential anti-senescence drug. Compared to senescent cells (control), senescent cells treated with R)-(+)-Limonene displayed a lower percentage of senescent cells (Fig. 7K), lower expression levels of LMα4 and p-p38, and similar expression levels of T-p38 and MMP-9, via IB (Fig. 7L). Considered together, these results demonstrated that the p38 MAPK pathway interacted with LMα4 expression in HLE B-3 cells, and that p38 MAPK activation induced by senescence was related to LMα4 upregulation.

### TGF-β1 and LMα4 are elevated in cataractous ALCs with senescence

**Figure 8:**
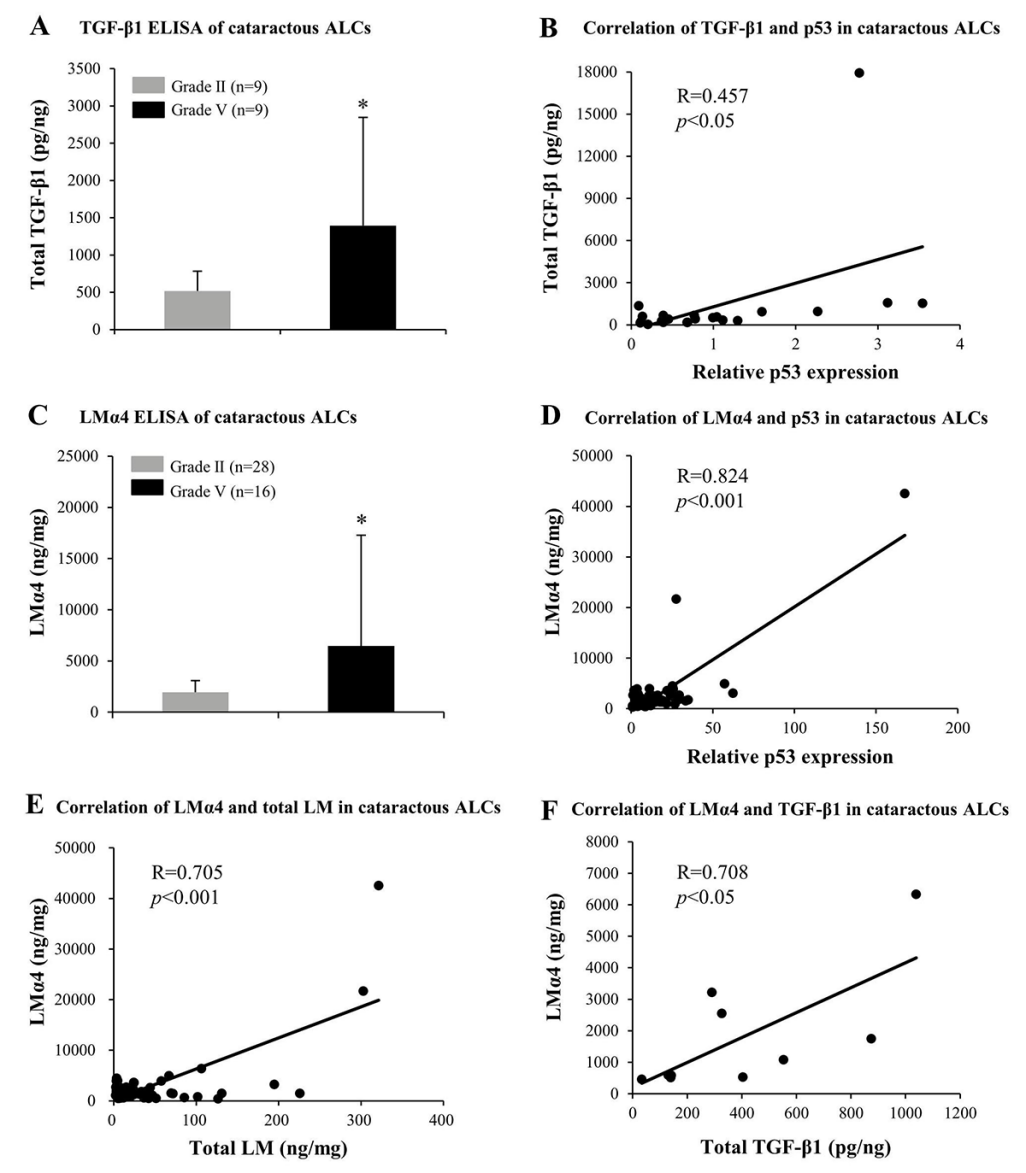
**Elevated TGF-β1 and laminin α4 (LMα4) in the cataractous anterior lens capsules (ALCs) with senescence.** Human ALCs, graded before surgery for cataract severity by the Emery-Little Classification System of nuclear opacity grade, were obtained from the anterior surface of cataractous lenses during surgery. **(A)** Total TGF-β1 in human ALCs with ARC of grade II and grade V groups, as detected by ELISA. The figure depicts a Pearson correlation between TGF-β1 expression and senescence (n = 22) **(B)**. **(C)** LMα4 subunit in human ALC groups groups with ARC grades II and V, as detected by ELISA. The figure depicts a Pearson correlation between LMα4 expression and senescence (n = 64) **(D)**. **(E)** Association between LMα4 and total LM in ALCs with ARC (n = 60). **(F)** Association of LMα4 and TGF-β1 in ALCs with ARC (n = 9). Data were shown as mean ± SD and were analyzed using the Wilcoxon Rank Sum Test. *, *p*<0.05.

In order to further confirm the possible contribution made by LMα4, MMP-9, and TGF-β1 to the pathogenesis of ARC, protein expression levels of LMα4, MMP-9, and TGF-β1 in cataractous ALCs were analyzed via ELISA. In grade V cataractous ALCs, total TGF-β1 (*p*<0.05); (Fig. 8A) and LMα4 (*p*<0.05); (Fig. 8C) were significantly higher than those in grade II ALCs, wherein their protein levels were also increased in a senescence-dependent manner (R=0.457 and 0.824, respectively); (Fig. 8B and 8D). However, MMP-9 was not detectable in cataractous ALCs (data not shown). In addition, in ALCs of ARC, LMα4 expression levels were positively correlated with those of total LM (R=0.705); (Fig. 7C) and TGF-β1 (R=0.708); (Fig. 7D), respectively.

### Summary of anterior lens capsular proteins and possible regulation mechanism in ARC

**Figure 9:**
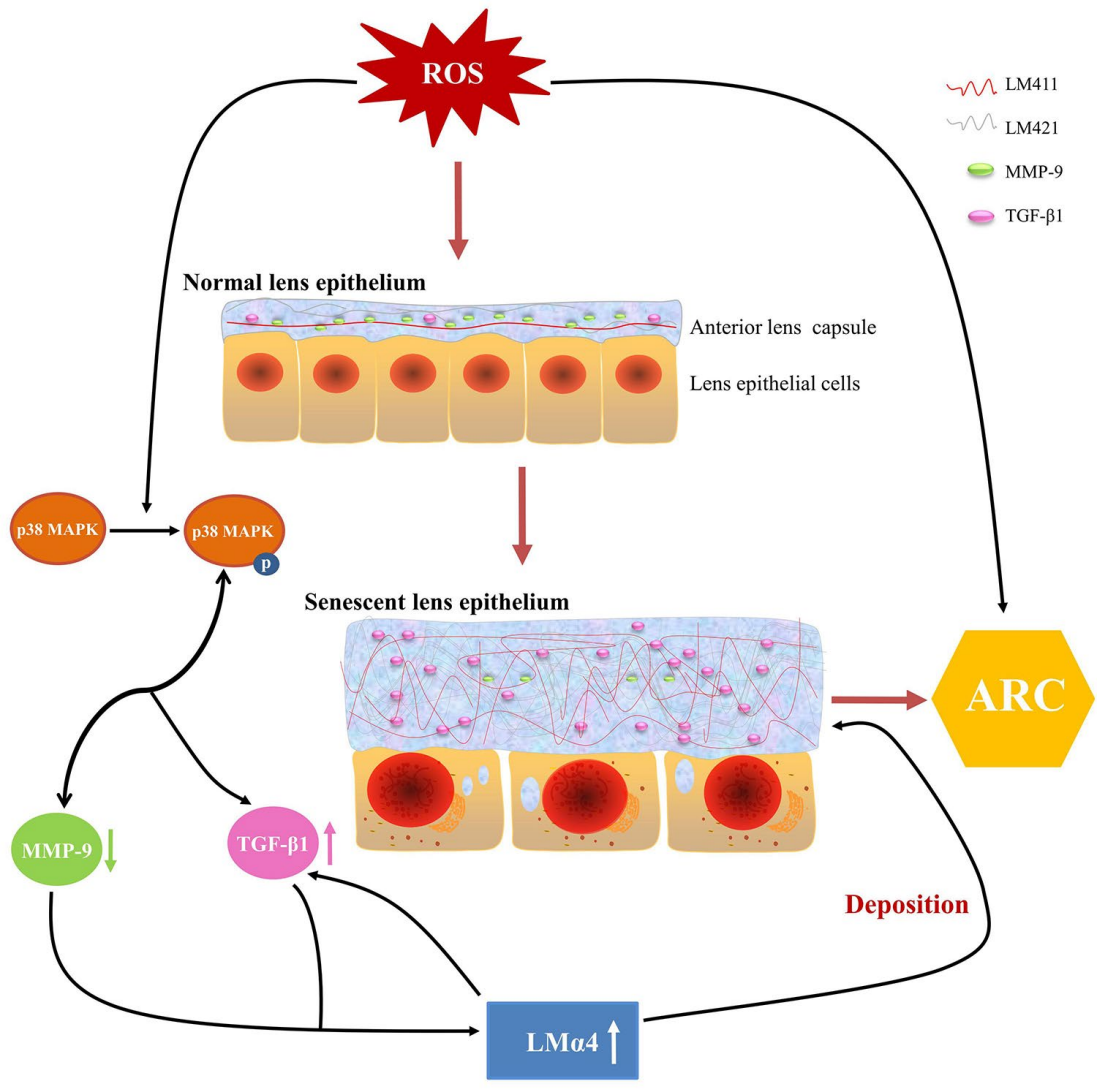
**Summary of anterior lens capsular proteins and possible regulatory mechanism in age-related cataract (ARC).** Schematic diagram of reactive oxygen species related senescent lens epithelial cells and senescent anterior lens capsules along with up-regulation of total LM, LMα4 and TGF-β1 and down-regulation of MMP-9.

Based on our experimental evidence, we proposed a possible mechanism for ARC development. ROS in aqueous humor increased with aging, stimulating p38 MAPK activation, followed by the downregulation of MMP-9 expression and upregulation of TGF-β1 expression, which leads to an excessive accumulation of total LM and LMα4 in senescent primary LECs, finally, such aberrant deposition of total LM, LMα4, TGF-β1 and MMP-9 in senescent ALCs results in ARC (Fig. 9). Taken together, our findings indicate novel pathological roles for TGF-β1, MMP-9 and LMα4 in ARC.

## DISCUSSION

The objectives of this study were to determine whether ALCs senescence influences the severity of ARC, as well as whether the capsular proteins, total LM, particularly LMα4, and their possible mediators, MMP-9 and TGF-β1, played a role in such a senescence response, thus contributing to the development of ARC.

Our observation that the severity of ARC increased with age, and the expression level of the senescence-related marker p53 in cataractous ALCs, suggested that senescence of ALCs promotes the development of ARC. However, the mechanisms underlying ALC changes in ARC during senescence are largely unknown. It is known that LMs are not only increased in the capsules of cataractous lenses [32,33] but also during senescence, which was associated with thickening of the BM [34]. These results are all consistent with the findings of the present study. In this study, we showed that the total LM expression was increased in higher grade (more severe) cataractous ALCs, and that such grade-related total LM expression is positively associated with the senescence of cataractous ALCs. Additionally, we found that except for LMα4, LMγ2, and LMγ1, which reportedly exist in the ALCs of transparent lenses [77], the ALCs of ARC were also positive for 3 other LM subunits, LMα2, LMα1, and LMβ3, and higher expression levels of these were also observed in senescent HLE B-3 cells and cell BMs compared to controls. Therefore, we contend that LMα2, LMα1, and LMβ3 subunits most likely contribute to cataract formation, although all of these were not detectable in ALCs of ARC via ELISA (data not shown). Nevertheless, it remains necessary to elucidate the association between LMα2, LMα1, and LMβ3 and ARC development in further studies.

LMα4 is a component of LM-8, which is composed of α4, β1 and γ1, and LM-9, which is composed of α4, β2 and γ1. It is found in various tissues of mesenchymal origin, endothelial BMs, and certain epithelial BMs [83–85]. Studies centered on the role of LMα4 in cataract formation are scant. The current study showed that either LM411 or LM421 was the predominant LM trimer in ALCs of ARC. We also found that excess LMα4 in the cataractous ALCs of higher grades and observed that LMα4 expression was positively associated with the senescence of cataractous ALCs. This suggested that LMα4 overexpression may contribute to the senescence of ALCs and ARC development. In addition to the relevance of LMα4 to ARC, it may also have certain important cell biological functions in LECs. The effect of LMα4 on cell adhesion, cell migration, angiogenesis, and differentiation has been well studied in cells other than LECs [86–88]. Results of the current study demonstrated that sheep anti-LMα4G may inhibit cell senescence, but may also increase cell migration abilities and cell viabilities in HLE B-3 cells. In addition, LMα4 siRNA reduced ATP1A1 expression in HLE B-3 cells, which were expressed minimally in differentiating lens cells [66,67], suggesting that LMα4 may affect LEC differentiation. Therefore, appropriate suppression of LMα4 may present a therapeutic basis for treating senescence or ARC. Nevertheless, it is felt that further studies may be necessary to determine whether LMα4 itself or the downstream mechanism represents the most viable target for such a treatment.

Age-related changes in MMP-9 expression are associated with many diseases. Some studies suggested that MMP-9 expression may increase with age [89] while others showed that it decreased with age [90,91]. Reportedly, silencing MMP-9 may induce cell senescence [92,93]. In addition, MMP-9 may process LM and latent TGF-β in some cells [43,44]. Therefore, we investigated the effect of MMP-9 on cell senescence as well as on LM and TGF-β1 expression. We found that senescence was associated with a decrease in MMP-9 expression in HLE B-3 cells and cell BMs. MMP-9 overexpression impeded cell senescence and senescence-associated total LM, LMα4 and TGF-β1 expression changes in senescent cells and cell BMs, whereas MMP-9 inhibition showed the opposite response. These suggested that impaired MMP-9 levels in senescent cells may contribute to BM protein remodeling that accompanies senescence. It remains to be investigated whether other contributing factors exist, as also how impaired MMP-9 triggers this process. Furthermore, we found that inhibition of p38 MAPK phosphorylation reversed the reduction in MMP-9 expression in cellular senescence. MMP-9 silencing in senescent cells further activated p38 MAPK phosphorylation. These findings indicate that activated p38 MAPK and impaired MMP-9 levels, form a vicious cycle, inducing abnormal protein expression of in senescent cells and cell BMs, and finally promoting the development of ARC. However, further investigation of the associations between MMP-9, p38 MAPK, LMs, and TGF-β1 in the development of ARC is felt to be necessary.

It has been reported that TGF-β1 triggers the progression of senescence [51,52]. Recently, an autocrine TGF-β1 signaling axis that induces cell senescence was found, where TGF-β1→TGF βRI→Smad1/5/8:Smad2→p53→p21→cell senescence [53]. The current study showed that TGF-β1 and TGF βRI may be key determinants of senescent characteristics and the extent of senescence in HLE B-3 cells. We provided evidence that TGF-β1 was highly expressed in senescent cell BMs and higher grade cataractous ALCs. SB431542, a TGF βRI inhibitor, significantly inhibited cell senescence, whereas active TGF-β1 RP significantly increased senescence-associated p21 expression. These findings suggested that cell senescence and p21 activation triggered by H_2_O_2_ were partly dependent upon TGF-β1 and TGF βRI. We identified a possible signaling axis that induces senescent phenotypes in HLE B-3 cells as follows: H_2_O_2_→TGF-β1→TGF βRI→p21→cell senescence. This may require further confirmation. Previous studies have shown that TGF-β1 may downregulate total LM mRNA expression in HLE B-3 cells [60]. However, it induces aberrant deposition of LMα2, LMα1 and LMβ1 in glomerular BMs [61]. Our results clearly indicated that SB431542, a TGF βRI inhibitor, significantly reduced the aberrant deposition of total LM, LMα4 and LMβ3 in senescent BMs. Active TGF-β1 RP triggers the accumulation of LMα4 and LMβ3 in HLE B-3 cells. The positive relationship between TGF-β1 expression and LMα4 deposition in cataractous ALCs suggests that TGF-β1 may promote the development of ARC by stimulating the aberrant deposition of LMα4 in ALCs.

Some studies have shown that p38 MAPK is involved in LM signaling [81], which is known to mediate apoptosis and senescence in cells. Inhibition of p38 MAPK decreased p16 and p21 levels in senescent cells [65], and may delay the progression of H_2_O_2_-induced opacification of lenses [94]. A recent study showed that SB203580 significantly reduced LMα4 expression in trophoblast cells [95]. The current study proved that inhibition of the p38 MAPK pathway significantly abated cell senescence and LMα4 expression. In addition, p-p38 was expressed at a higher level following senescence, and this was accompanied by the upregulation of LMα4 expression. Our findings indicated that the LMα4-MAPK pathway is likely to be responsible for senescence. However, LMα4 may be regulated via different pathways under varying conditions in HLE B-3 cells, requiring further explorations. Our results showed that SB203580 not only significantly reduced TGF-β1, LMα4 and LMα1 expression but also increased LMγ1, collagen 1α1 and MMP-9 expression levels in senescent HLE B-3 cells. These results indicated that SB203580 may prevent HLE B-3 cells from undergoing senescent changes in response to H_2_O_2_. Therefore, we speculate that p38 inhibitors may be exploited as a potentially useful tool for cataract prevention and may also be useful for treating existing cataracts.

R)-(+)-Limonene is a monoterpene commonly found in the essential oils of citrus fruits. It is widely used in soaps, foods, perfumes, chewing gum, and beverages as a flavoring agent and fragrance additive [96]. Many studies have demonstrated that R)-(+)-Limonene, which possesses powerful antioxidative properties, may eliminate oxygen free radicals and protect organisms from oxidative damage [97–99]. More importantly, it has been shown to reduce H_2_O_2_-induced ROS generation and cell apoptosis in LECs via the p38 MAPK pathway [100]. Therefore, we used (R)-(+)-limonene on HLE B-3 cells to further confirm the association of LMα4 accumulation and p38 MAPK phosphorylation with cell senescence. Our results showed that R)-(+)-Limonene reduced conditions leading to cell senescence, and the expression of LMα4, p-p38 in HLE B-3 cells, suggesting that R)-(+)-Limonene may eﬀectively prevent cell senescence by reducing H_2_O_2_-induced LMα4 overexpression and inhibiting H_2_O_2_-induced p38 MAPK phosphorylation. The eﬀects of R)-(+)-Limonene that attenuate cell senescence requires further studies in order to elucidate the underlying mechanism(s). Based on the current study we hypothesized that attenuating the expression of LMα4 in senescent cells and BMs may provide a new target for cataract prevention and treatment.

In conclusion, our data indicated that excess deposition of LMα4 in cataractous ALCs, possibly caused by the down-regulation of MMP-9, up-regulation of TGF-β1 and activation of p38 MAPK signaling during senescence, contributes to the development of ARC. LMα4 and its regulatory factors show potential as targets for drug development for prevention and treatment of ARC.

## MATERIALS AND METHODS

### Ethics statement

All experiments were performed with the approval of the Internal Review Board of Harbin Medical University and were conducted in accordance with the principles of the Declaration of Helsinki.

### Human ALCs and protein lysates

Human ALCs obtained from 300 individuals with ARC, aged 50–97 years old, were collected between 2016 and 2018 at the 1st Affiliated Hospital of Harbin medical University (Harbin, China). Informed consents were obtained from all donors and their family members. Human ALCs were obtained by a single ophthalmologist from anterior surface of cataractous lenses during surgery, using the central circular capsulorhexis method [101]. Following careful dissection from cataractous lenses, ALCs were temporarily stored in PBS at 4°C. Subsequently, the capsules were washed with PBS twice and stored at 80°C prior to protein extraction. All obtained ALCs were free from other ocular diseases and were graded for cataract severity using the Emery-Little Classification System of nuclear opacity grade before surgery [102,103]. Human cataractous ALCs were lysed using cold radioimmunoprecipitation assay (RIPA) buffer [50 mM Tris-HCl (pH 7.5), 150 mM NaCl, 1% Triton X-100 and 1% protease inhibitor cocktail from Sigma Aldrich; Merck KGaA, Darmstadt, Germany] overnight at 4˚C. Finally, the supernatant was maintained as a human cataractous ALC lysate following centrifugation at 14,000 *x g* at 4°C for 20 min.

### Bicinchoninic acid assay

Protein concentrations were measured using a Bicinchoninic acid assay kit (Beyotime Biotechnology, Shanghai, China) according to the manufacturer’s instructions.

### ELISA

The total LM levels in human cataractous ALCs, HLE B-3 cells and cell BMs were assayed using commercially available LM ELISA kits in accordance with the manufacturer’s recommendations (E-EL-H0128c, Elabscience, Wuhan, China). The antibody used in this kit was polyclonal antibodies (pAbs) against all kinds of LMα, LMβ and LMγ subunits. LMα4 subunit levels in human cataractous ALCs were examined by using commercially available LMα4 ELISA kit (CSB-EL012728HU, CUSABIO, Wuhan, China). In addition, total TGF-β1 levels in human cataractous ALCs, HLE B-3 cells and cell BMs were assayed using a commercially available TGF-β1 ELISA kit (E-EL-H0110c, Elabscience).

### HE staining and immunohistochemistry staining of human ALCs

ALCs were careful dissected from cataractous lenses, embedded in an embedding medium [OCT compound (4583, Sakura Finetek, Torrance, USA)] and stored at -80°C. Frozen human cataractous ALC tissues were transversely sectioned at 5-µm thickness, mounted on glass slides, fixed, subjected to HE and IHC staining. For HE staining, human cataractous ALCs were stained with hematoxylin and eosin (5 min and 2 min, respectively, at room temperature), and examined under a light microscope (Olympus Corporation, Tokyo, Japan). For IHC of LMs, cataractous ALCs were incubated with 3% H_2_O_2_, blocked in 10% normal goat serum for 20 min at room temperature, and incubated with rabbit anti-LM antibodies (1:200; ab11575, Abcam Company, Cambridge, UK) for 60 min at room temperature. Next, the samples were treated with secondary antibodies and color development was performed using 3, 3’-diaminobenzidine (DAB) as the chromogen. Under identical experimental conditions, normal rabbit IgG (1:200; sc-2025, Santa Cruz Biotechnology, Dallas, TX, USA) were used as the isotype control. Staining was visualized with a light microscope (Nikon TE300, Nikon Corporation, Tokyo, Japan), and images were captured using a digital camera and associated software (SPOT Basic™ image capture software; cat. no. SPOT53BE; SPOT Imaging, a division of Diagnostic Instruments, Inc., Sterling Heights, MI, USA).

### Antibodies

The antibodies used in this study include rabbit pAbs against LMα4, LMβ3, LMγ2, TGF-β1, MMP-9 and p53 (1:1000; C13067, C13071, C30224, C0340, C30044 and B0530, Assay Biotechnology Company, San Francisco, California), rabbit pAbs against LMγ1 and GLB1 (1:3000; ab69632 and ab128993, Abcam Company), rabbit pAb against MMP-9 (1:200; sc-10737, Santa Cruz Biotechnology), rabbit pAbs against p21 and ATP1A1 (1:2000; 10355-1-AP and 14418-1-AP, Proteintech, Chicago, USA), rabbit pAb against LMα5 (1:2000; E-AB-31903, Elabscience), rabbit monoclonal antibody (mAb) against collagen 1α1 (1:2000; ab138492, Abcam Company), and mouse mAbs against LMα4, LMα3, LMα2, LMα1, LMβ2 and LMβ1 (1:200; sc-130540, sc-13586, sc-55605, sc-74418, sc-133241 and sc-17763, Santa Cruz Biotechnology).

### Immunoblotting

Protein levels in the human cataractous ALC lysate, HLE B-3 cell lysate and HLE B-3 BMs were analyzed via IB as described previously [77]. Briefly, antigen sources including protein lysates of human cataractous ALCs, HLE B-3 cells, and HLE B-3 cell BMs were mixed with 2X sample buffer, boiled for 2 min and subjected to SDS-polyacrylamide gel electrophoresis (SDS-PAGE). Separated proteins were then transferred to a PVDF membrane (Millipore, Darmstadt, Germany). After blocking with 5% skim milk in Tris-buffered saline containing 0.05% Tween 20 (TBS-T), membranes were incubated overnight with the aforementioned primary antibodies diluted in solution 1 (TOYOBO, Osaka, Japan) at 4°C. After being washed with TBS-T, the membranes were incubated with HRP-conjugated goat anti-mouse IgG (1:5000) or HRP-conjugated goat anti-rabbit IgG (1:5000) diluted by solution 2 (TOYOBO) for 1 h at room temperature. Finally, the antibody–antigen complex was visualized using an Enhanced Chemiluminescent (ECL) kit (Beyotime Biotechnology).

### Immunoprecipitation-immunoblotting

All subsequent procedures were performed at 4°C, unless otherwise stated. sample containing 360 µg of human ALC lysate from 10 patients with ARC was incubated overnight on a shaker with protein G agarose (Beyotime Biotechnology) and anti-LMα4 rabbit pAb. Equal concentrations of normal rabbit IgG were used as the isotype control for IP. Precipitates, collected by centrifugation at 3,000 *x g* at 4°C for 2 min, were washed thrice with PBS containing 0.5% Triton X-100, and eluted from protein G agarose by boiling with 4X sample buffer for 2 min. Proteins in the supernatant were separated via SDS-PAGE and detected by IB, as mentioned above.

### HLE B-3 cell culture and preparation of cell lysate

Human LEC HLE B-3 line were obtained from the American Type Culture Collection (Manassas, VA, USA) and cultured in low-glucose DMEM (Hyclone; GE Healthcare Life Sciences, Logan, UT, USA) containing 10% fetal bovine serum (FBS) (Gibco; Thermo Fisher Scientific, Inc., Waltham, MA, USA), 100 U/ml penicillin and 100 µg/ml streptomycin (Beyotime Biotechnology) at 37°C in a humidified atmosphere with 5% CO_2_. Cells were seeded in 10-cm dishes or 6-well plates (Nunclone, Thermo Scientific, CA) treated with various factors. Following removal of the culture supernatant, cells were washed twice with PBS and lysed with cold RIPA buffer at 4°C for 30 min. Following centrifugation at 14,000 *x g* at 4°C for 20 min, the supernatant was harvested and retained as cell lysate.

### Assessment of cell viability

Effect of H_2_O_2_ on the viability of HLE B-3 cells was assessed using 3-(4,5-dimethylthiazol-2-yl)-2,5-diphenyltetrazolium bromide (MTT) assay. H_2_O_2_ was freshly diluted in cultured DMEM. Brieﬂy, HLE B-3 cells were seeded into a 96-well plate at a density of 1.0× 10^5^ cells/ml and a volume of 100 µl per well and cultured with various H_2_O_2_ concentrations (0, 200, 300, 400, 500 and 600 µM) in 100 µl of culture medium for 96 h. Thereafter, 20 µl of MTT [5 mg/ml in phosphate-buffered saline (PBS, pH 7.4)] was added to each well and the plate was incubated at 37°C for a further 4 h. Following removal of 150 µl of the supernatant per well, the plate was added with dimethylsulfoxide (DMSO) at 150 µl/well and vortexed at 700 *x g* at room temperature for 10 min. Next, optical density (OD) was measured at 570 nm using a microplate reader (BioTek, Winooski, VT). The following formula was used: relative percentage of cell viability = (OD_570_ of the experimental sample/ OD_570_ of the control group) x 100%. The control cells were not exposed to H_2_O_2_.

### Premature senescence model and SA-β-gal staining

To produce the H_2_O_2_ induced senescence model, cells were exposed to various concentrations of H_2_O_2_ (200, 300, 400, 500, and 600 µM) for 96 h. Cells were then fixed in solution with PBS containing 2% formaldehyde and 0.2% glutaraldehyde for 10 min at room temperature. After rinsing with PBS, the cells were incubated with freshly prepared SA-β-gal staining solution (pH = 6.0) containing 1 mg/ml X-gal (Solarbio Science & Technology Co., Beijing, China), 200 mM potassium ferricyanide, 200 mM potassium hexacyanoterrate and 100 mM MgCl_2_ for 16 h at 37°C. SA-β-gal positive and negative cells (cells with greenish color) were counted in 10 fields of each well under a light microscope (Nikon Corporation) and photographed with a camera (Olympus Corporation). The percentage of SA-β-gal positive cells percentage was estimated as the mean number of positive cell/ the mean number of total cells.

### BM preparation using HLE B-3 cells

HLE B-3 BMs grown on 6-well plates following various treatments were harvested, as described previously [77]. Briefly, HLE B-3 cells were washed with PBS 4 times, following which 0.03% ammonia and 0.1% Triton-PBS were used to remove HLE B-3 cells completely. Finally, HLE B-3 BMs remaining on the bottom of the plates were washed 4 times with double distilled water and harvested with 100 µl of 4X sample buffer at room temperature or 200 µl RIPA buffer on ice. A 20-40 μl aliquot of human cataractous ALCs, as well as HLE B-3 cell BMs was sufficient to obtain clear polypeptide bands using SDS-PAGE (10%-gel) stained with CBB (Beyotime Biotechnology).

### Immunofluorescence assay

HLE B-3 cells and BMs grown on 24-well plates following various treatments were fixed with 4% paraformaldehyde solution containing 0.18% Triton X-100 for 30 min at 4°C. After rinsing thrice PBS, specimens were incubated with PBS containing 2% goat serum (Shang hai yuan mu, Shanghai, China) for 1 h at 37°C. Fixed cells or BMs were then incubated for 1 h at 37°C with the rabbit pAbs against p21 (1:500), LMα4 (1:100), LMβ3 (1:50) and MMP-9 (1:100; 10375-2-AP, Proteintech). After washing thrice with PBS, cells or BMs were incubated with FITC-anti-rabbit IgG (1:250, A11008, Life Technologies, New York, USA) for 30 min at room temperature. Next, the cells were washed thrice with PBS and incubated with DAPI (1:5000, C0060, Solarbio, Beijing, China) in PBS for 2 min at room temperature. However, this step was not performed for BMs. Finally, the cells or BMs were visualized using a Leica DMRA immunofluorescence microscope and Leica software (Leica Microsystems, Wetzlar, Germany).

### Senescent HLE B-3 cells transfection

The pCDNA3.1-MMP-9, MMP-9 siRNA, LMα4 siRNA, and their respective control were chemically synthesized or purchased from GenePharma Co. (Shanghai, China) and transfected into senescent HLE B-3 cells using Invitrogen Lipofectamine™ 2000 transfection reagent (Thermo Fisher Scientific). The following senescent groups, senescent-pCDNA3.1, senescent-pCDNA3.1-MMP-9, senescent-siRNA, and senescent-MMP-9/ LMα4 siRNA, were established. Detailed information regarding MMP-9 siRNA, LMα4 siRNA, and their controls is listed as follows: MMP-9 siRNA (5'-GCGCUGGGCUUAGAUCAUU-3'); LMα4 siRNA (5'-CAGGGAUUUAUGCAGAAAU-3'); control-siRNA (5'- UUCUCCGAACGUGUCACGU-3'). For pCDNA3.1-MMP-9 transfection, 2µg MMP-9 plasmid and 10 µl Lipofectamine™ 2000 were added to 500 µl serum-free culture medium and incubated at room temperature for 20 min following mixing. The mixture was added to senescent cells. The supernatant was replaced 6 h later with fresh 10% FBS culture medium. Following incubation for 48 h at 37°C, transfected cells/ BMs were subjected to SA-β-gal staining, IB, or immunofluorescence assay, performed as mentioned above. For MMP-9 or LMα4 knockdown, the senescent HLE B-3 cells were seeded in 6-well plates and transfected with 100 pM MMP-9 or LMα4 siRNA or control siRNA using Lipofectamine™ 2000 and a protocol similar to that for plasmid transfection.

### Reagent administration

Details of reagents administration are presented (Table 1). MMP-9 inhibitor I (sc-311437, Santa Cruz Biotechnology) was dissolved in DMSO to obtain a 5 mM stock solution, and applied to cells 12 h before addition of H_2_O_2_ (final concentration, 5 µM). SB431542 [TGF-β RI inhibitor, (HY-10431, MedChemExpress, New Jersey, USA)] and LY2109761 [TGF-β RI/II inhibitor, (HY-12075, MedChemExpress)] were dissolved in DMSO to prepare a 20 mM and 10 mM stock solution, respectively. SB431542 and LY2109761 were applied to cells (final concentration, 10 µM and 5 µM, respectively) which had been exposed to 400 µM H_2_O_2_ for 24 h. TGF-β1 RP (APA124Hu01, USCN Business Co., Ltd., Wuhan, China) was dissolved in PBS to prepare a 10 µg/ml stock solution and applied to cells at 15 ng/ml for 96 h. SB203580 (S8307-1MG, Sigma Aldrich; Merck KGaA, Darmstadt, Germany), a cytokine-suppressive anti-inflammatory drug, was used as a p38 MAPK inhibitor in this study. It was also dissolved in DMSO at 30 mM to prepare a stock solution. The cells were pretreated with SB203580 at 30 μM for 4 h before the addition of H_2_O_2_. R)-(+)-Limonene with a purity of 97% was purchased from Sigma Aldrich and diluted using culture medium prior to use. Cells were pretreated with R)-(+)-Limonene at 1000 µM for 4 h prior to addition of H_2_O_2_.

**Table 1:** Reagents.

Reagent	Cat nos.	Dilution		Source
		Stock concentration (solution)	Working concentration (culture medium)	
MMP-9 inhibitor I	sc-311437	5 mM (DMSO)	5 µM	Santa Cruz Biotechnology
SB431542 (TGF-β RI inhibitor)	HY-10431	20 mM (DMSO)	10 µM	MedChemExpress
LY2109761 (TGF-β RI/II inhibitor)	HY-12075	10 mM (DMSO)	5 µM	MedChemExpress
TGF-β1 RP	APA124Hu01	10 µg/ml (PBS)	15 ng/ml	USCN Business Company
SB203580	S8307-1MG	30 mM (DMSO)	30 μM	Sigma Aldrich
R)-(+)-Limonene	183164	-	1000 µM	Sigma Aldrich

### Cell proliferation assay

The effect of sheep anti-LMα4G (AF7340, R&D Systems, Abingdom, UK) on the proliferation of HLE B-3 cells was analyzed via a Cell Counting Kit-8 (CCK-8) assay. The CCK-8 assay was performed according to the manufacturer's instructions. Briefly, HLE B-3 cells were seeded in 96-well plates in medium containing 2 μg/ml of sheep anti-LMα4G and cultured for 96 h. Then, 10 μl of CCK-8 (Dojindo, Japan) reagent was added to the preparation following which it was incubated at 37°C for 4 h. Absorbance was measured at 450 nm using a microplate reader (BioTek, Winooski, VT). Cell proliferation in the presence of 2 μg/ml sheep IgG was defined as 100% control. The relative percentage of cell proliferation was calculated as follows: (OD_450_ of the experimental sample/ OD_450_ of the control group) x 100%. All experiments were performed in triplicate and repeated at least 3 times.

### Migration assay

The effect of sheep anti-LMα4G (R&D Systems) on the migration of HLE B-3 cells was evaluated via a migration assay. HLE B-3 cells, treated with 2 μg/ml of sheep anti-LMα4G for 48 h, were wounded by scratching with 1 ml tips. After scratching for 0 h and 48 h, images of cells were captured via camera (Olympus Corporation, Tokyo, Japan). The relative percentage of cell migration ability was calculated as follows: (cell migration distance of the experimental sample/ distance of the control group) x 100%.

### Statistical analysis

Statistical analyses were performed using SigmaPlot 12.0 (Hulinks, Inc., Tokyo, Japan). One-way ANOVA, Wilcoxon Rank Sum Test and Paired t-test were used to analyze the differences between experimental groups. Statistical significance was set at P < 0.05.
